# Supplementary material for: The interactome of intact mitochondria by cross-linking mass spectrometry provides evidence for coexisting respiratory supercomplexes
Source: Mol Cell Proteomics. 2017 Dec 8;17(2):216–32. doi: 10.1074/mcp.RA117.000470 (PMC5795388; doi:10.1074/mcp.RA117.000470)
Supplement: Supplemental Data [file supp_RA117.000470_133922_0_supp_23978_fzffwf.zip › spectra_annotation/mito_spectra_annotation/382-1-7-1-18-1.pdf]

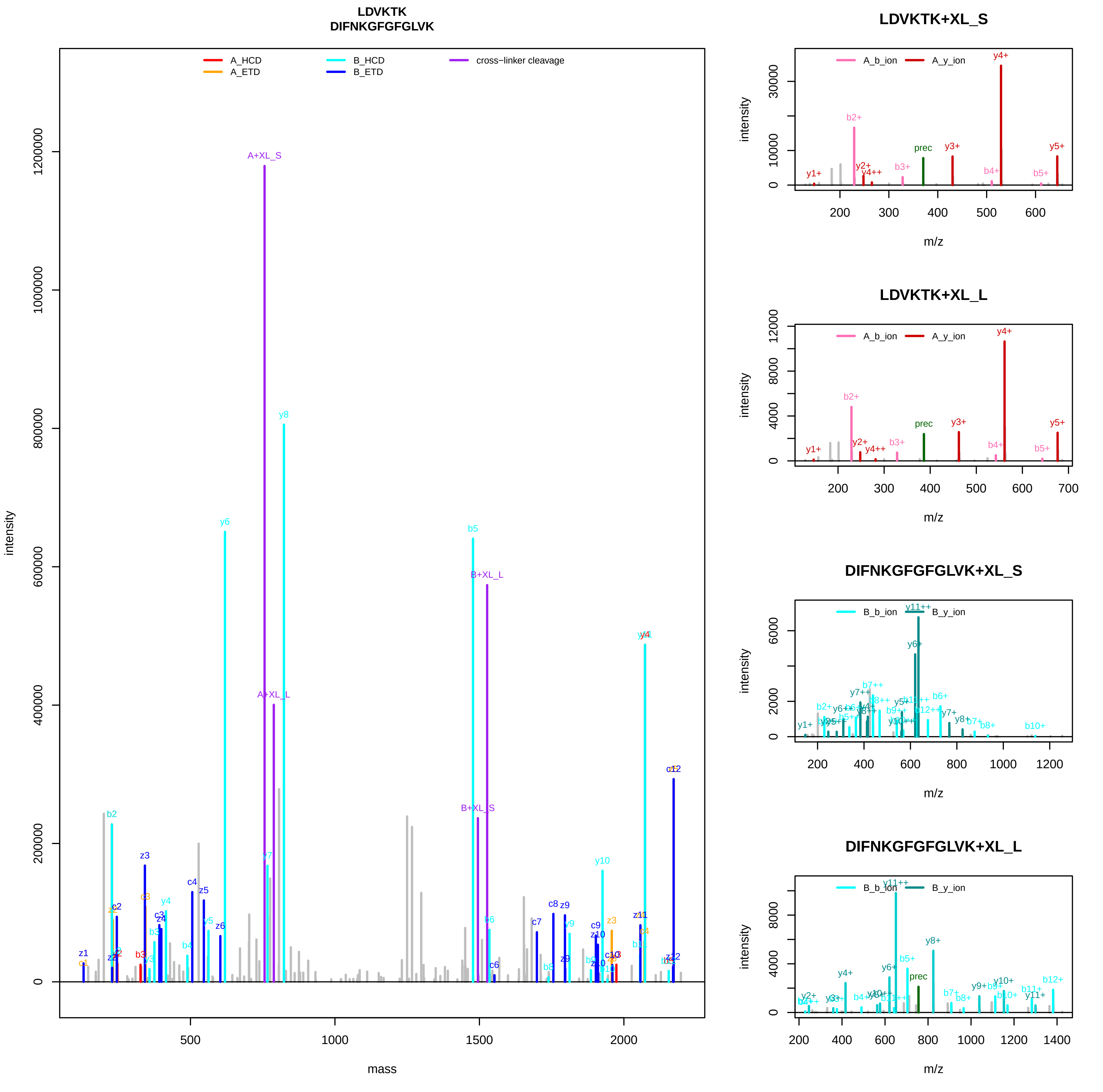

LDVKTK
DIFNKGFGFGLVK
A_HCD
A_ETD
B_HCD
B_ETD
cross−linker cleavage
intensity
mass
0
200000
400000
600000
800000
1000000
1200000
500
1000
1500
2000
A+XL_S
A+XL_L
B+XL_S
B+XL_L
y8
y6
b5
y10
y11
c12
b2
z3
c4
z5
c8
z9
LDVKTK+XL_S
LDVKTK+XL_L
DIFNKGFGFGLVK+XL_S
DIFNKGFGFGLVK+XL_L
A_b_ion
A_y_ion
B_b_ion
B_y_ion
y4+
b2+
prec
y3+
y5+
y11++
y6+
y8+
m/z
